# Supplementary material for: Evolving autonomous learning in cognitive networks
Source: Sci Rep. 2017 Dec 1;7:16712. doi: 10.1038/s41598-017-16548-2 (PMC5711912; doi:10.1038/s41598-017-16548-2)
Supplement: Supplementary file 1 — Supplementary Material [file 41598_2017_16548_MOESM1_ESM.pdf]

Supplementary Information for:
Machine Learned Learning Machines

Leigh Sheneman
Department of Computer Science and Engineering,
Michigan State University
BEACON-Center for the Study of Evolution in Action,
Michigan State University

Arend Hintze
Department of Integrative Biology Michigan State University
Department of Computer Science and Engineering,
Michigan State University
BEACON-Center for the Study of Evolution in Action,
Michigan State University

September 25, 2017

1 Feedback Gate

A feedback gate is very similar to a probabilistic logic gate, in that it has inputs and outputs and a probability table that defines the likelihood that an input gets mapped to an output at ever given update (See Figure 1 1). However, it also possesses two additional connections, one to detect when positive feedback is given, and one for negative feedback (See Figure 1 red and green wires going into the gate). In our specific example here, we assume that at the first update the gate did, it received an input of 0 for A and 1 for B. It then used the probability table and returned an output of 1 (See Figure 1 2). In the probability table we marked the value that mapped the input 0,1 to the output 1 in blue. Technically, all probabilities in the row for that input are responsible, but here we call only the probability in blue the responsible one. It should be higher if that mapping was advantageous, and lower if the decision of the gate was bad or had negative consequences. In step 3 the gate now receives a positive feedback signal, and no negative signal otherwise (See Figure 1 3). Consequently, the formerly marked blue probability needs to be increased indicated by the upward facing arrow in the probability table (0.7 becomes 0.8). Because each row in the table is summing to 1.0 in order to ensure that all entries remain probabilities, we need to re-normalize the row, and thus the other values need to be decreased (light gray probability with an arrow facing down. After feedback is computed, new inputs need to be processed. Here the gate receives a 1 for input A and a 0 for input B. This leads to a new output of 0, which identifies a new probability in the probability table, highlighted in blue (See Figure 1 3 blue highlight).

At the next update (See Figure 1 4), the gate now receives a new negative feedback, but no positive one. This means that the probability remembered (blue) was too high and needs to be decreased. The arrow facing down in the dark blue section of the table. Similarly, due to normalization, the other values in that row need to be increased, indicated by the upwards facing arrow in the light gray box. Again, after feedback was applied, the gate still needs to map the newly received input (1,1) to an output, and remembers the responsible probability (blue).

This principle would only allow for immediate feedback. When implemented A memory queue is added to remember the sequence of all responsible probabilities. The depth of the queue is evolvable and when feedback is applied, all probabilities in the queue become modified (positively or negatively depending on the nature of the feedback). The magnitude of the change is

also evolvable. The elements of the queue are processed sequentially for oldest to youngest, and after each step of the sequence the probability matrix becomes normalized again.

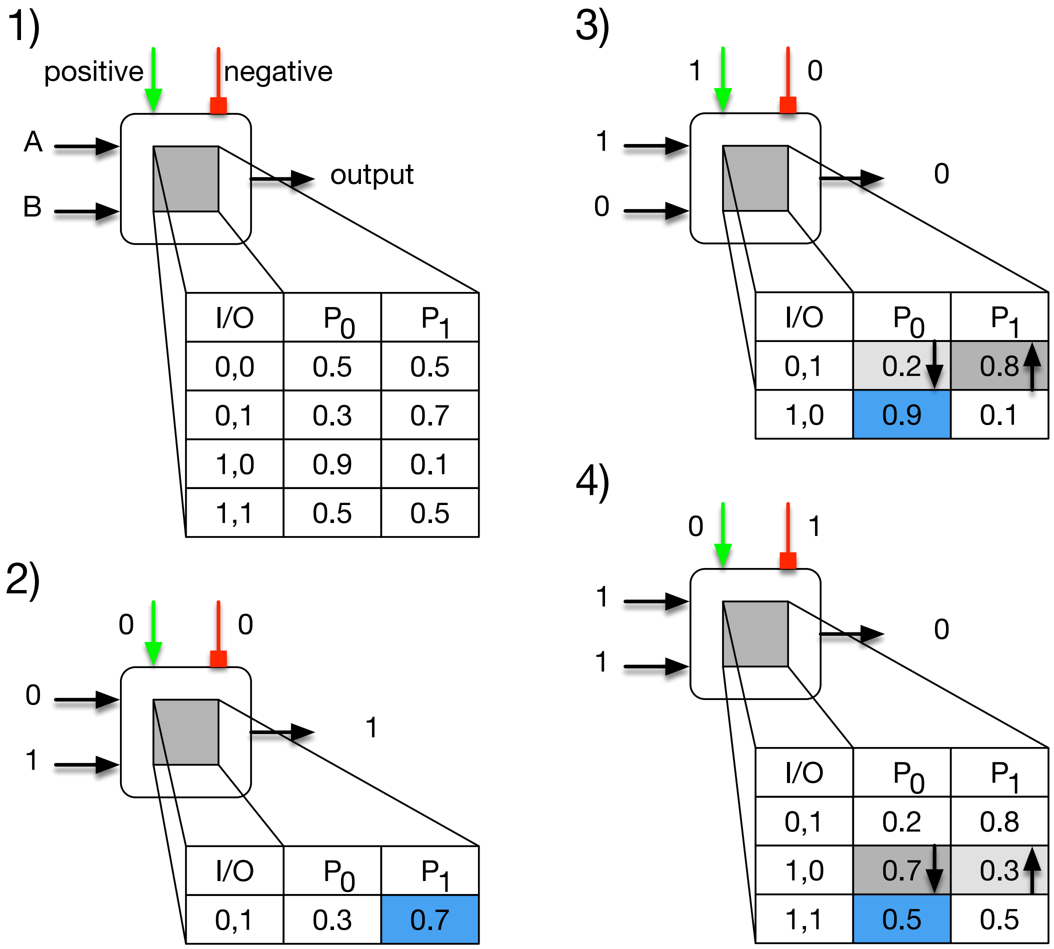

Figure 1: Functionality of a feedback gate. See the text for an explanation.

2 Minimum Performance Distribution

For each evolutionary experimental condition, we recorded the minimum number of times each of the 300 agents reached the goal across all 24 maps. We see that minimum number of times an agent reaches the goal has a left-skewed distribution in all three treatments, indicating that learning the task is non-trivial.

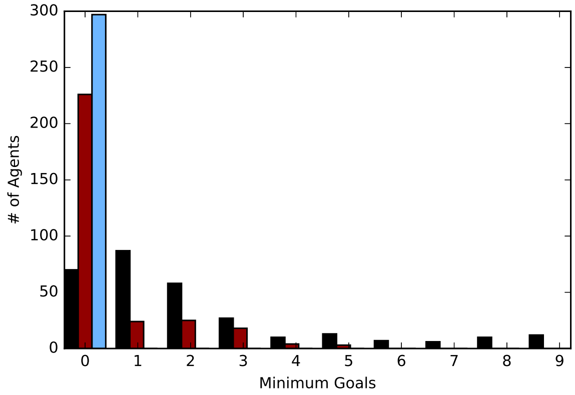

Figure 2: Minimum Performance Distribution for three experimental conditions. Black all gates allowed, blue probabilistic gates allowed, red only deterministic gates.

3 Feedback Integration

Agents that were evolved using feedback gates have the potential to change their probability table within their lifetime. By computing the difference between the tables from each feedback gate at birth and after 512 updates, we find that indeed these tables change over their lifetime, but this difference neither increases or decreases over evolutionary time (see Figure 3). The difference was computed using the root mean square method. In addition, we find only a very weak correlation between the amount of change and performance (see Figure 4).

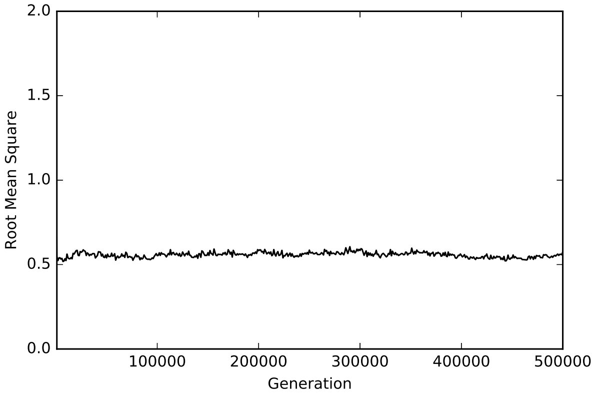

Figure 3: Root mean square difference between probability tables at birth and after learning is over for each generation on the LOD.

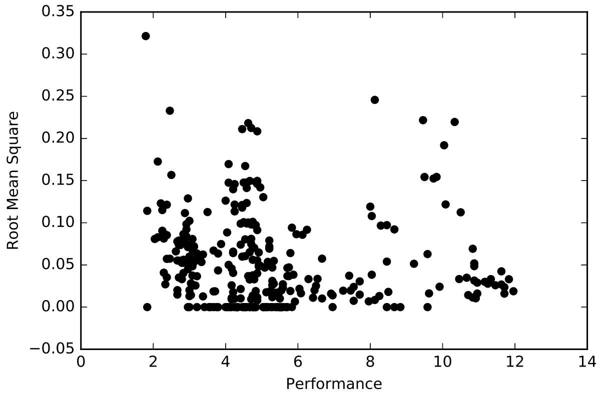

Figure 4: Correlation of change in root mean square to performance at the end of evolution.

4 Computational Networks

When we compare the behavior of the best performing agent using feedback gates, with that of the best performing agent that does not use feedback gates, we find one very striking difference. To exemplify this we show the best performing agent without feedback gates (see Figure 6) to evolve a complex network of deterministic gates (shown with square boxes) and seem to make use of the hidden nodes of the Markov Brain. While the best performing agent that has feedback gates (see Figure 5), seems to write to the hidden states less and both output nodes receive a signal from the feedback gate (seen in the octagon).

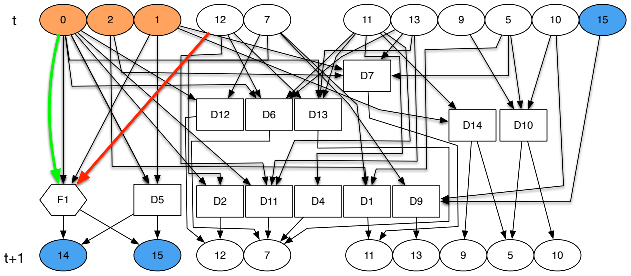

Figure 5: Computational network of best performing agent using feedback. Input nodes are orange, output nodes are blue, hidden nodes are white. Positive feedback connections are green arrows, and negative feedback are red arrows. Logic gates are rectangular boxes, while the feedback gate is an octagon. The top row of nodes represents the states at time point t, while the bottom row of nodes represents the internal states of the Markov Brain at time point $t + 1$.

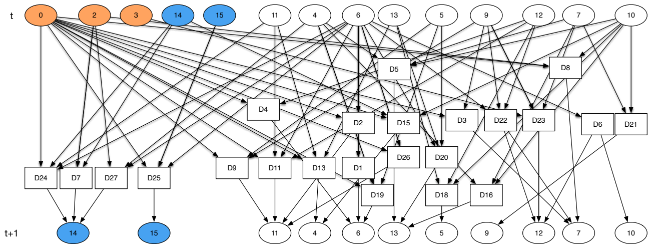

Figure 6: Computational network of best performing agent not using feedback. Input nodes are orange, output nodes are blue, hidden nodes are white, logic gates are rectangular boxes. The top row of nodes represents the states at time point t while the bottom row of nodes represents the internal states of the Markov Brain at time point $t + 1$.

5 Performance Plots

Each image (see Figure 7) depicts an environment where the cells are labeled using a gray arrow, pointing towards the optimal path. Dark gray boxes are walls or obstacles in the environment. The red arrows indicate the orientation and location an agent had during its life. Once agents reached the goal, they were put back to a random location 23 steps away from the goal. The left two images, labeled A and C, depict the path in environment 16, and the right two images, labeled B and D, show environment 21. We see that either evolved agent moves on a straight line without attempting to take turns when they move on a straight line of forward facing arrows. However, the agent using feedback gates makes less mistakes when it comes to turns, whereas the agent not using feedback gates struggles to find the correct direction at every turn and reaches the goal less often.

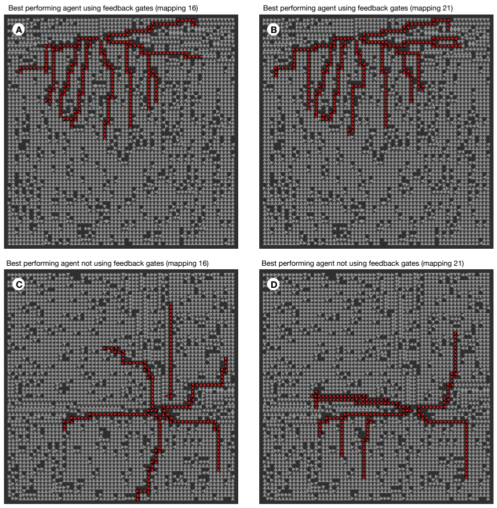

Figure 7: Path comparison of the best performing feedback agent versus best performer without feedback.

6 Average Updates to Goals

For the top 50 performing agents using all three types of gates–deterministic, probabilistic, and feedback gates– we recorded the number of updates it takes to get to the first five goals (see Figure 8). This allows us to estimate the progression of learning. We find that the time to reach the first goal takes about 118 steps, to the next one only 54, and finally this trend stabilizes at about 50 steps to the goal.

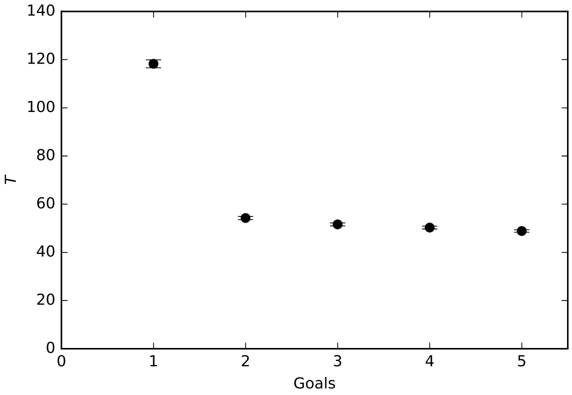

Figure 8: Average updates required to reach first five goals.

7 Learning a Single Map

To create a fair comparison between Q-learning and MBs we evolved 300 replicates of MBs to solve one map for 5,000 generations. Here we plot the mean performance over evolutionary time for agents on the LOD (see Figure 9). Agents where able to match the performance of the Q-learners less than 400 generations.

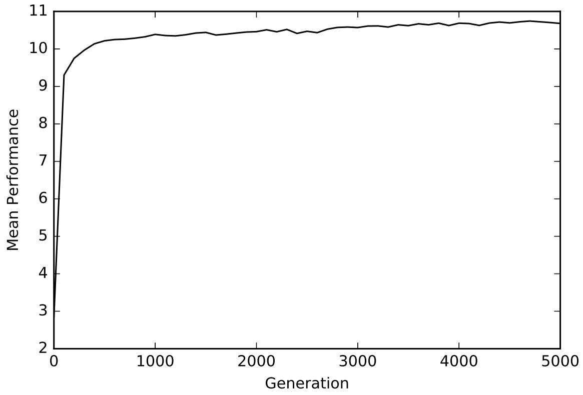

Figure 9: Performance over evolutionary time when evolved on a single map.

8 General function of feedback learning agents

Reconstructing the set of logic instructions for each agent is simple, however the algorithm implemented by an agent remains epistemically opaque (See Marstaller 2010). All computations happen in parallel and representations about concepts are spread over nodes instead of being neatly compartmentalized. This makes it impossible to distill a narrative. However, general statements like the number of nodes or the number of gates are possible. Also, different evolutionary experiments might result in different types of solutions, which further complicates the attempt to create a narrative for the function of an agent. When trying to understand the evolved agents we found at least one remarkable design feature. Very much like the agents controlled by a heuristic, feedback gates preferred to receive positive feedback from the sensor indicating that the agent now faces forward. It seems reasonable to do that since actions that turn the agent to be facing forward are always good.
